# Morphological Engineering of Filamentous Fungi: Research Progress and Perspectives

**DOI:** 10.4014/jmb.2402.02007

**Published:** 2024-03-26

**Authors:** Zhengwu Lu, Zhiqun Chen, Yunguo Liu, Xuexue Hua, Cuijuan Gao, Jingjing Liu

**Affiliations:** 1 College of Life Sciences, Linyi University, Linyi 276000, P. R. China; 2 Shandong Fufeng Fermentation Co., Ltd., Linyi 276600, P. R. China

**Keywords:** Filamentous fungi, morphological engineering, mycelial morphogenesis

## Abstract

Filamentous fungi are important cell factories for the production of high-value enzymes and chemicals for the food, chemical, and pharmaceutical industries. Under submerged fermentation, filamentous fungi exhibit diverse fungal morphologies that are influenced by environmental factors, which in turn affect the rheological properties and mass transfer of the fermentation system, and ultimately the synthesis of products. In this review, we first summarize the mechanisms of mycelial morphogenesis and then provide an overview of current developments in methods and strategies for morphological regulation, including physicochemical and metabolic engineering approaches. We also anticipate that rapid developments in synthetic biology and genetic manipulation tools will accelerate morphological engineering in the future.

## Introduction

Filamentous fungi are a group of microorganisms with a highly developed mycelium, characterized by diverse morphology, multinucleation, multicellularity, and high chromosome number compared to bacteria and yeast. Most of them belong to the taxonomic divisions *Ascomycota* and *Basidiomycota*, which are found in different natural habitats, but the taxonomic discussion is still ongoing [1, 2]. Currently, filamentous fungi are one of the important expression systems for the production of microbial fermentation products [3], which are widely used in the food, textile, medical, and chemical industries and have great commercial application value [4, 5]. For example, filamentous fungi, especially *Ascomycetes* and *Basidiomycetes*, are known to produce an extraordinary range of colors, including several chemical classes of pigments, such as melanins, azaphilones, flavins, phenazines, and quinines [6]. Compared to other conventional sources, industry is more focused on filamentous fungi, which can be easily grown in the laboratory and provide the possibility of large-scale production [7]. Filamentous fungi can secrete a wide variety and large amounts of enzymes, including glycosylases, proteases, phytases, pectinases, and peroxidases [8-10], which can degrade polymers in organic wastes, such as (hemi)cellulose and starch, into small molecules as energy and carbon sources. Thus, filamentous fungi also play an important role in nature by degrading organic wastes [11, 12].

The enormous secretion capacity of fungi has already been used by industry for decades to produce organic acids and polymers, represented by citric acid, polylactic acid, gluconic acid, itaconic acid and lactic acid, as potential alternatives to petroleum-based plastics or raw materials [13]. Of these, citric acid has been commercially produced using *Aspergillus niger* for almost a century, generating significant economic value [14]. In addition, filamentous fungi are productive strains of some important antibiotics, such as penicillin [15]. Thus, filamentous fungi have wide applications in the food, chemical, agriculture, and pharmaceutical industries, and research on them plays a catalytic role in the emerging global bio-economy, circular economy, and healthcare.

Despite the obvious advantages of working with filamentous fungi, it can sometimes seem difficult to obtain higher production levels. In traditional food fermentation processes, such as wine making, filamentous fungi tend to carry out their metabolic activities in the form of mycelia [16]. In large-scale industrial production, submerged fermentation is typically used to efficiently produce large quantities of product using aerated-stirred bioreactors [17]. However, in submerged fermentation, filamentous fungi are often associated with highly non-Newtonian properties and variations in fungal morphology. Bacterial and yeast growth can be easily described by the duplication of single cells, whereas filamentous fungi undergo a complex morphological evolution from conidia to hyphae to dispersed mycelia or distinct particles [18]. Variations in fungal morphology are not only difficult to detect and control, but are often accompanied by changes in the rheological, mass transfer, and mixing properties of the fermentation broth [19]. This makes the fermentation process complex and difficult to control, which in turn affects the synthesis of the target product and the efficiency of the fermentation process. The dispersed hyphae increase the viscosity of the medium, which affects mass transfer, and nutrient distribution is extremely uneven. The hyphae will wrap around the impellers of the fermentation tank, causing clogging and diffusion into the sampling and overflow lines [20]. However, compared to other microorganisms, apart from the greater difficulty of gene editing and transformation, the main problem seems to be the cultivation process itself. The existence of these problems affects the growth and development as well as the metabolism and product production of filamentous fungi. Therefore, studies on the morphology of filamentous fungi under submerged fermentation are of great importance. This paper first reviews the mechanism of mycelial development in filamentous fungi, and then summarizes the methods for regulating mycelial morphology, in which the importance of morphometabolic breeding for regulating mycelial morphology is outlined in detail.

## Mycelial Morphogenesis of Filamentous Fungi

Filamentous fungi are characterized by their ability to form highly polarized hyphae; no other cell type, with the exception of neurons and pollen tubes, exhibits such an extreme degree of polarized growth [21]. Due to their multicellular nature, filamentous fungi maintain a more complex morphology and characteristic chemoattractant mechanisms than unicellular fungi [22]. In recent years, new insights have been gained into the mechanisms of cell growth and morphological development of filamentous fungi, but they are still not well understood [23].

**Fig. 1:**
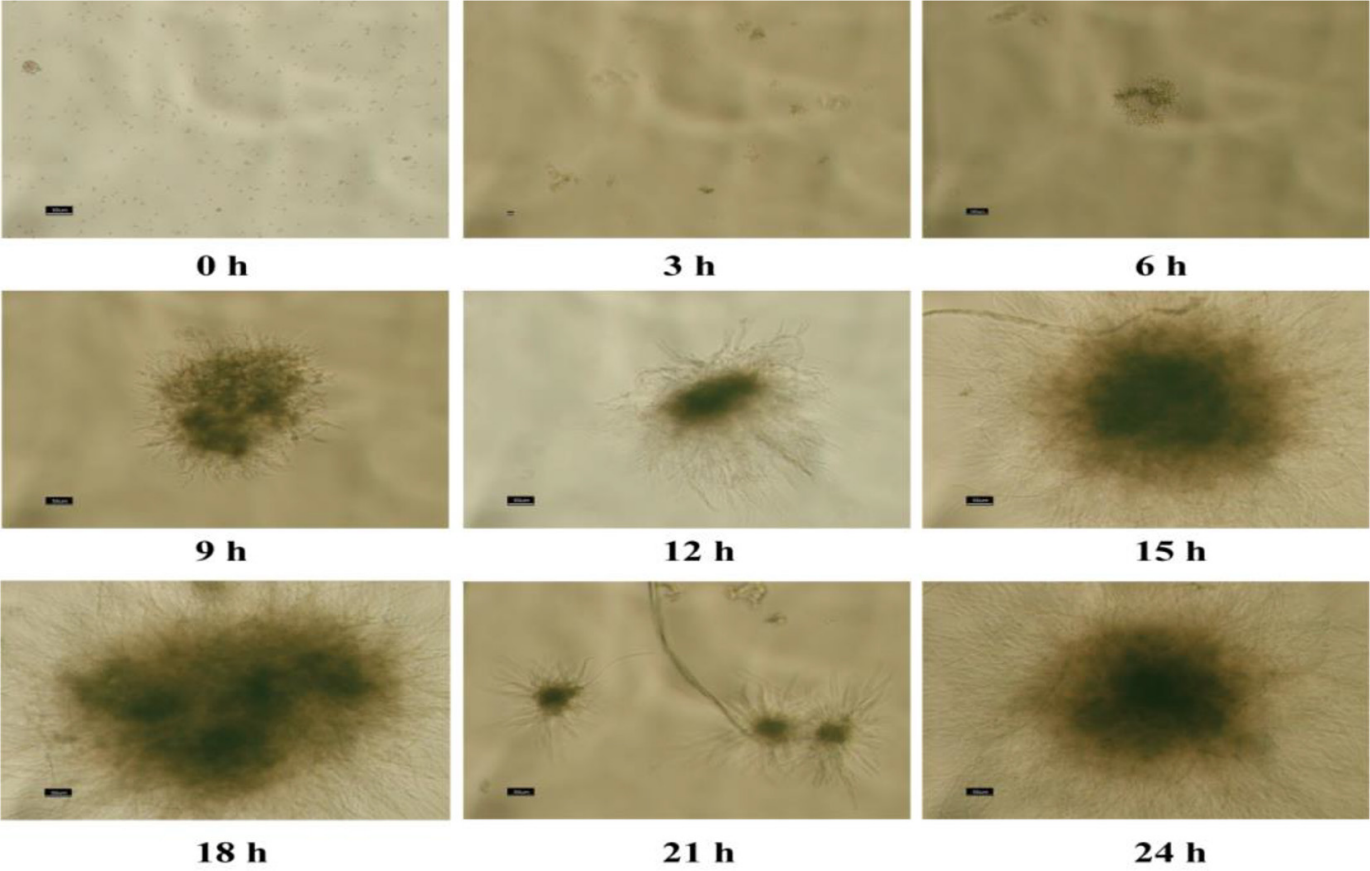
The process of spore germination under submerged culture conditions.

Filamentous fungi reproduce by means of spores, the first stage being spore germination [21]. Under suitable nutrient conditions, the spores break metabolic dormancy, swell, and gradually synthesize cell walls as water enters the cells. Next, myosins and morphogens recruit the actin cytoskeleton at specific sites to establish polarity, which is continuously maintained to produce a highly polarized germ tube that grows into a hypha [24]. The hallmark of hyphae is the growth of mycelial cells by apical extension [25], when proteins associated with the cell membrane, extracellular hydrolases, and cell wall synthesis are 'packaged' into Golgi vesicles and transported along microtubules and the actin cytoskeleton to the tip. The vesicles accumulate at the mid-apical position and bind to the exocyst protein complex, anchoring them to the plasma membrane [26]. Mycelial polarity is mediated by cell-end-marking proteins on the plasma membrane, with the tip inserting into the new membrane by exocytosis and taking up membrane binders and soluble material into the cell by endocytosis. Polar cells extend in an oscillatory manner, with pulses of Ca^2+^ injection coordinating the cycle of actin polymerization, cytosolic exocytosis, and tip extension. The process of mycelial extension exerts pressure on the newly synthesized cell wall, and this internal pressure is essential for the continued growth of the mycelium [27]. Thus, control of cell wall integrity is a fundamental aspect of mycelial growth and viability. On the other hand, the delivery of cell wall synthetase from the vesicle to the tip must be balanced with the secretion of enzymes for extracellular nutrient acquisition. As growth continues, the mycelium divides across the wall by forming septa. Once a mature hypha has formed, mycelial growth enters the second-phase branching [21]. The mycelium forms branches at the apical or lower insertion zone, and different mycelia are able to fuse together, eventually forming a network of cells called the mycelium. As the mycelium matures, the secondary cell wall gradually thickens and the mycelium reproduces asexually by producing conidia. The process of dispersed spores germinating and gradually forming a pellet under submerged fermentation conditions is shown in Fig. 1.

While traditional solid-state cultivation has clear benefits in some types of processes, there are many technical limitations when considering industrial applications [28]. On the other hand, deep liquid fermentation is more widely used in industry, and a great deal of research in this area has already been conducted. Compared with solid-state fermentation, submerged fermentation has significant advantages, such as easy scale-up, simple parameter control and processing, short fermentation cycle, and low labor intensity and cost savings. Moreover, this culturing process is becoming a major form of industrial biotechnology for producing target fungal metabolites, such as industrial enzymes, antibiotics, organic acids, and pigments [29, 30]. However, the variable morphology of filamentous fungi under submerged fermentation conditions is one of the main constraints to their productivity. During submerged cultivation in bioreactors, the morphology of filamentous fungi can be one of three morphological forms, namely dispersed mycelia, clumped aggregates, or mycelial pellets (including rough and smooth pellets) [18, 19, 31]. Fungal morphology can be strongly and intricately determined by environmental conditions and inherent molecular or genetic biology. After inoculation of conidia into the liquid medium, they aggregate due to electrostatic, salt bridge or hydrophobic interactions between surface polysaccharides or proteins, resulting in the aggregation of multiple single spores to form a mycosphere [32]. Apparently, pellets can reduce the viscosity of fermentation broth and improve mechanical strength, but slow growth and metabolism in the interior regions of large particles may limit the formation of the target product due to poor oxygen diffusion. In contrast, dispersed mycelium grows rapidly and has no limitations in nutrient transport. Some disadvantages of the dispersed growth state compared to pellets include higher medium viscosity, limited mass heat and momentum transfer between gas and liquid, and increased tolerance to shear stress, which has affected the yield and productivity of target products in bioreactors [33].

## Research Progress on Morphological Control of Filamentous Fungi

### Physicochemical Methods for Control of the Morphology

**Fig. 2:**
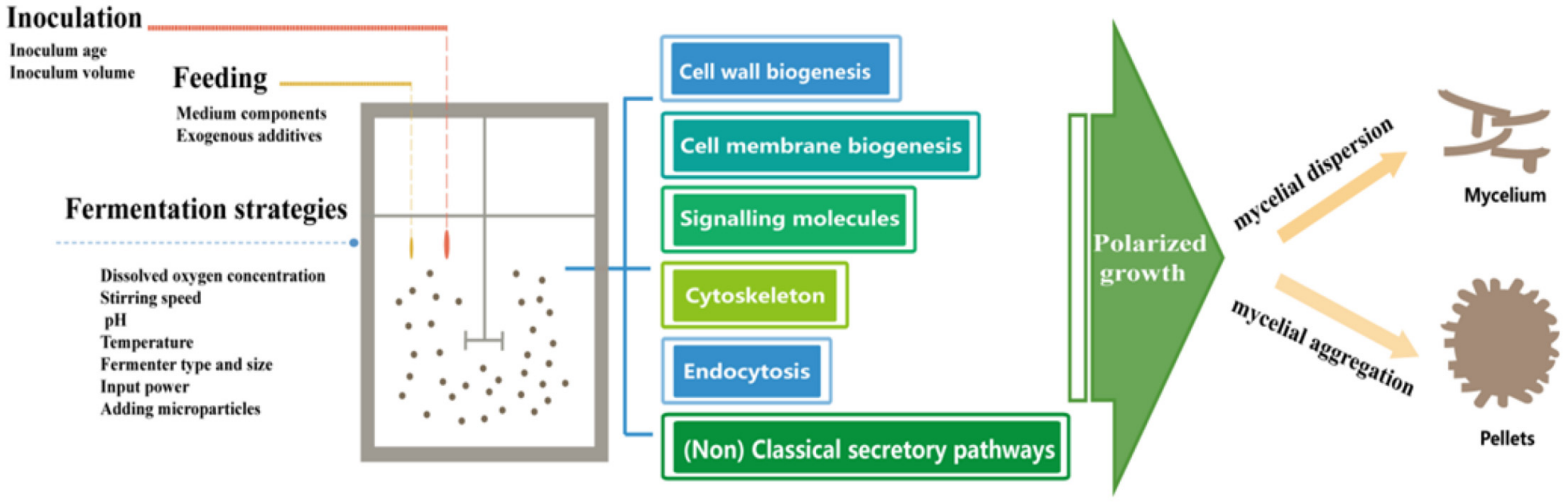
Mechanisms of influence on morphology during submerged fermentation of filamentous fungi.

Fungal morphology has been shown to vary with changes in nutrients and environmental conditions during fermentation. Factors that have been reported to influence the morphology of filamentous fungi include inoculum age, inoculum volume, medium components, exogenous additives such as surfactants, dissolved oxygen concentration, stirring speed and stirring paddle shape, pH, temperature, fermenter type and size, and input power (Fig. 2) [5, 34].

The results of many studies have shown that morphology is one of the important factors influencing the ability to synthesize products, and the traditional means of regulating morphology include changing the variables in the fermentation environment or adding particle enhancers (Table 1) [35, 36]. Bioreactor configuration also plays a crucial role in fungal morphology, as it affects solid-liquid mixing, aeration, agitation, shear stress, etc. [28]. The design of bioreactors is usually based on the mechanisms of mass transfer and shear stress, as they determine the availability of oxygen, diffusion of nutrients, cellular integrity, etc. [37, 38]. However, the targeted design and processing of equipment is more time-consuming and labor-intensive than engineering the fungal morphology to match the required conditions. Furthermore, bioprocesses that can be more easily adapted to existing infrastructures are more likely to be implemented in large-scale industrial production [28].

*A. niger* is an excellent host for protease and amylase production, and the effect of morphology on enzyme production has been studied to some extent [5]. The addition of silicate and alumina particles of different sizes and concentrations can also result in many different morphologies of *A. niger*. Analysis of morphogenetic images during culture showed that the addition of particles disrupted the aggregation of conidia and formed loose mycelium more conducive to enzyme secretion [48]. The addition of talc microparticles at concentrations of 1 g/l, 3 g/l, and 10 g/l prior to inoculation reproducibly produced a variety of morphological structures. The most relaxed morphology was obtained with the addition of 10 g/l talc microparticles and the highest β-fructofuranosidase activity was observed [50]. Different morphologies of the oleaginous fungus *Mortierella isabellina* were observed by addition of different concentrations of magnesium silicate microparticles. Significantly higher lipid content (0.75 g lipid/g cell biomass) and lipid yield (0.18 g lipid/g glucose consumed) were achieved in freely dispersed mycelia than in pellets, and the by-product malate was suppressed [53]. In addition to the addition of particles, there was also a significant increase in protease activity released by *A. niger* when the mycelial morphology was broken down from pellet to filamentous during fermentation using carboxymethylcellulose [41]. In addition to the loose morphology being more conducive to the production of some enzymes, oxygen appears to be necessary for enzyme production, but pellets are known to limit the diffusivity of oxygen, substrates, and products. The pure filamentous morphology was found to be more favorable for the production of chitosan during the fermentation of *Trichoderma reesei*, although the change in morphology did not have a significant effect on the synthesis of ethanol [56].

Compared with the filamentous or flocculent form, the aggregation of spores into pellets during submerged fermentation can effectively reduce the viscosity of the fermentation broth and facilitate separation, which is advantageous for the production of products such as organic acids, fatty acids, and secondary metabolites [32]. Currently, 99% of the world's citric acid is derived from the fermentation of *A. niger* pellets [5]. A study of the relationship between the morphological development of *A. niger* and the level of spore inoculation found that at a spore inoculum concentration of 10^4^-10^5^ particles/ml, *A. niger* can easily form pellets under liquid fermentation conditions, which is more favorable for citric acid production. As the spore concentration increases, the dissolved oxygen concentration in the fermentation broth decreases rapidly, the length and branching frequency of the mycelium increases significantly, the development continues in a dispersed mycelial form, and the production of citric acid decreases significantly [35]. The study found that some nutrients such as soy peptone and calcium carbonate favored the formation of smooth pellets during the culture, whereas the addition of metal ions had a significant negative effect on pellet formation. Adding potato dextrose broth (PDB) and calcium carbonate can significantly increase the yield of organic acids produced by *Rhizopus oryzae*, as its production of lactic acid and fumaric acid was increased from 32.0 g/l and 21.5 g/l to 65.0 g/l and 31.0 g/l, respectively [45]. Pellet diameter was also found to have a significant effect on cell productivity. The diameter of pellets in fermenters can be up to several millimeters, while dissolved oxygen and other nutrients in the fermentation broth can only penetrate about 200 μm into the surface of the mycelial spheres [20], causing activity within the pellets to decrease dramatically, or even become inactive [35]. In polygalacturonase production by *A. sojae*, polygalacturonase activity was increased by 74% and biomass by 40% when the diameter of the pellets was between 0.05 and 0.76 cm by controlling the nutrient conditions, stirring speed, and spore concentration [44]. *Aspergillus* sp. showed the highest yield of biomass and glucosamine at a diameter of 2.15 mm, and an increase or decrease in diameter was detrimental to the synthesis of products [47]. The titanate particles (TiSiO_4_, 8 mm) could bind tightly to the cells to form core-shell particles. The smaller particles and core-shell structure reduced the thickness of the biomass layer, and the resulting loose internal particle structure also allowed for higher mass transfer and penetration depth, resulting in a 3.7- and 9.5-fold increase in the activity of furanosidase and glucoamylase, respectively, in the growth medium of *A. niger* with increasing titanate content [36].

Although optimization of fermentation conditions can achieve some effective control of morphology, it is not yet possible to accurately predict the most favorable state for the production of the target product, and therefore requires considerable effort in process design.

### Metabolic Engineering of Morphology

In addition to physicochemical conditions, the morphology of filamentous fungi during fermentation is regulated by several genes encoding proteins that alter morphology by controlling cell size, mycelial polarity, or the spacing of intercellular septa (Table 2) [59]. In 2001, Professor Jens Nielsen, an international authority on metabolic engineering, pioneered metabolic engineering of morphology by studying the effect of mycelial aggregation on metabolic pathways and target product synthesis at the molecular level [59, 60]. Comparative genomics is a powerful tool for uncovering gene-level information on superior variants, and with advances in DNA sequencing technology, more fungal genetic information is being annotated and published [61]. This achievement combined with advances in gene editing and RNA technology has accelerated metabolic engineering-based breeding in fungi [62].

In 1999, Bocking *et al*. used UV and nitrite mutagenesis to obtain a highly branched mycelial mutant strain of *A. oryzae* with reduced fermentation broth viscosity and increased glycosylase production [69]. Traditionally, fungal morphogenesis and secondary metabolism were thought to be independent, but genome sequencing revealed mutations in the gene encoding the methyltransferase (LaeA) of the low-viscosity *Penicillium chrysogenum* mutant, which is involved in the synthesis of the heterotrimeric velvet complex in filamentous fungi [70]. Currently, LaeA mutations have been applied to modify various filamentous fungi, which can simultaneously activate natural product synthesis and regulate cell morphology [71]. Through genomic comparative analysis, the high-yield protein variant of *A. niger* was found to have differences in gene sequences encoding cell wall synthesis compared to the original strain, including cell signaling, chitin synthesis, and β-1,3-glucan synthesis.

Chitin is a major structural component of the cell wall of filamentous fungi, and the final reaction in the chitin biosynthetic pathway is catalyzed by chitin synthase (*chs*), which has become a high-profile target for the investigation of the factors that influence the morphology, yield, and productivity of the fungus in submerged fermentation [60, 72]. Based on amino acid sequence homology, the *chs* enzyme family can be divided into seven classes (I to VIl), with different fungal species expressing different numbers of *chs* genes [73]. By blocking the expression of the gene for chitin synthase B (*chsB*) in *A. oryzae*, the apical growth rate of *A. oryzae* mycelium was 88% slower than that of the wild type, while the frequency of mycelial divergence increased by 188%. In addition, the modified *A. oryzae* mycelium did not aggregate into clumps during submerged fermentation, which facilitated overall control of the fermentation process, although α-amylase production did not increase [60]. Using RNAi technology to reduce the expression levels of *chsC* and *chs4*, genes encoding chitin synthase in *A. niger* and *P. chrysogenum*, respectively, to obtain dense hyphae and highly branched morphology, citric acid and penicillin yields were increased by 40% and 27-41%, respectively [64, 67].

In addition to the direct effects of the *chs* genes, the regulation of chitin biosynthesis is also determined to varying degrees by a number of other factors, including transcription factors, mitogen-activated protein kinase (MAPK), and calcium [74]. Transcriptomics revealed a sophisticated defense system in *A. niger* that employs at least three transcription factors, including RlmA, MsnA, and CrzA, to protect against cell wall stress [75]. Of these, CrzA is a calcium-signaling transcription factor that can bind to the promoter region of the *chs* gene, thereby regulating chitin biosynthesis and ultimately affecting mycelial aggregation patterns [76]. Thus, the calcium/calcineurin signaling pathway and the transcription factor CrzA are promising targets for biotechnological manipulation of fungal growth, development, and stress resistance. In *T. reesei*, deletion of the crzA gene resulted in a hyperbranched phenotype, which was accompanied by increased secretion of haemicellulases [77]. In addition to the calcium signaling pathway, the cAMP/PKA signaling pathway also influences growth and metabolism in filamentous fungi [5]. In this pathway, activation of a GPCR causes an adenylate cyclase to catalyze the conversion of ATP to cAMP, which then activates cAMP-dependent protein kinase A (PKA). Activated PKA phosphorylates various target proteins, including transcription factors, resulting in their entry into the nucleus and modification of gene expression [78]. In *A. niger*, overexpression of the PKA signaling subunit PkaC resulted in a more compact colony morphology [79]. In addition to overexpression, the *ACY1* and *PKAC1* genes coordinate light, filamentous growth, and cellulase gene expression in *T. reesei*, providing a way to simultaneously titrate morphology and cellulase expression [80].

Transcriptomic sequencing techniques (RNA-seq) and microarray gene expression profiling have revealed potential candidate genes for optimizing fungal morphology in different industrial processes. Moreover, comparative analysis of transcriptomic information between high-yielding strains and wild fungi during fermentation revealed significant differences in transcript levels of genes involved in morphology and cell growth, including typical and atypical secretion pathways, cytoskeletal components, endocytosis, cytosolic action, and cell wall and cell membrane biosynthesis [81]. In addition, proteins encoded by more than 2000 genes, including various signaling pathways that drive and control the aforementioned subcellular processes, are involved to some extent in the growth and development of filamentous fungi [82]. These studies suggest that the altered morphology of the fungus may be a result of cellular differentiation, accompanied by a response of the fungus to changes in the cellular microenvironment. Chen *et al*. controlled the optimal range of the total volume of pellets in a unit volume of fermentation broth (V value) by expressing the cytokinin-related proteins CDC14, CDC20, and CDC45, and controlled the expression intensity of CDC14 for L-malic acid production to 120-130 mm^3^/ml, so that the yield of L-malic acid was increased to 142.5 g/l in the fermenter [68]. The *gul1* gene encodes a putative mRNA-binding protein, and transcriptome analysis revealed that a number of genes encoding cell wall remodeling enzymes and hydrophobins were differentially expressed in the Δ*gul1* strain [83]. Smaller clumps of mycelium and a significantly lower viscosity of the fermentation broth were observed when *gul1* was disrupted in *Trichoderma reesei*, and cellulase production was improved by 22% compared to the parental strain [83]. A titer of 235.8 g/l L-malic acid was produced by *T. reesei* Δ*gul1*, which was a significant increase compared to the titer of 170 g/l of the original strain [84].

## Perspectives

Filamentous fungi in submerged fermentation conditions are diverse and sensitive to changes in environmental conditions, thus affecting the synthesis of target products and the efficiency of the fermentation process, which is one of the important factors affecting economic efficiency. Therefore, the study of the formation and control of fungal morphology is important for the stable control of the fermentation process and the realization of large-scale production.

The traditional means of controlling morphology, including physicochemical measures such as altering fermentation environment variables or adding particulate enhancers, can control morphological stability to some extent. However, the study of the fermentation process is hampered by the inability to accurately predict the optimal state of cell growth and target product production. While optimized bioreactor design is one way to achieve higher production targets, it is also time-consuming and labor-intensive, so strains and fermentation processes that can be more easily adapted to existing infrastructure are more likely to be implemented in large-scale industrial production. With the advancement of genomics and transcriptomics, the formation mechanism of filamentous fungal morphology under specific fermentation conditions has been intensively studied, and morphology optimization and design at the genetic level has become possible [85]. The genome size of filamentous fungi is several times larger than that of bacteria, the trophic cells are mostly diploid, and the mechanism of nonhomologous recombination makes gene targeting much more difficult, with low transformation efficiency and few available screening markers [86]. It is therefore more difficult to manipulate genes in filamentous fungi than in prokaryotes. The development of synthetic biology over the last few years is expected to overcome existing research bottlenecks [87]. For example, the CRISPR/Cas9 and CRISPRa systems have recently been introduced into filamentous fungi to explore the potential of target genes [88]. From *A. oryzae* and *T. reesei* to *A. niger* and *A. nidulans*, CRISPR/Cas9-based systems have become versatile platforms for precise genome editing, and great progress has already been made in the production of valuable metabolic products. In addition, fundamental tools for genome minimization have now been developed and are expected to reduce the genome complexity of filamentous fungi [89-91]. The combination of processes such as promoter sequence optimization, multi-copy expression of genes, transcription factors and post-transcriptional modifications gives filamentous fungi great potential in the sustainable bioeconomy.

Although it is becoming possible to control fungal morphology at the molecular level, there are still some issues that need to be considered and resolved in order to take full advantage of the excellent cell factories of filamentous fungi. For example, when using cheap substrate as a carbon source, a loose morphology is more favorable for hydrolytic enzyme secretion, but not necessarily for product synthesis, so how to achieve a balance between the two, or dynamic regulation of morphology? As fermentation conditions change, morphology-related genes are selectively expressed, so how can precise control be achieved at different stages? Also, there are many types of filamentous fungi, including septate and non-septate mycelium, mononuclear and multinuclear cells, so can a universal solution be applied to different types of industrial strains? With more researchers focusing on basic and applied fungal research, these challenges will be overcome and more excellent filamentous fungi will be applied to industrial production.

## Figures and Tables

**Table 1:** Advances in physicochemical methods to regulate the morphology of filamentous fungi.

Strains	Products	Regulatory parameters	Optimal morphology	Reference
*A. oryzae*	a-Amylase	pH	Filamentous	[39]
*A. niger*	Citric acid	Agitation	Little pellets	[40]
*A. niger*	Protease	Supplement carboxymethylcellulose	Filamentous	[41]
*A. terreus*	Lovastatin	Agitation and aeration	Smaller pellets	[42]
*A. niger*	Glucosamine	Spore inoculum level	Pellets	[35]
*A. oryzae*	Recombinant lipase	Feeding with maltose	Increase in hyphal diameter	[43]
*A. sojae*	Polygalacturonase	Concentrations of maltrin and corn steep liquor, agitation speed and inoculation ratio	The diameter of pellets between 0.05 and 0.76 cm	[44]
*Rhizopus oryzae*	Lactic acid	Addition potato dextrose broth and calcium carbonate	Small smooth pellets	[45]
*Caldariomyces fumago*	Chloroperoxidase	Microparticle-enhanced cultivation	Particles of 42 mm in diameter	[46]
*Aspergillus* sp.	Glucosamine	Pellet size, working volume, agitation rate and stimulating factor	pellet	[47]
*A. niger*	Glucoamylase and fructofuranosidase	Addition of microparticles	Freely dispersed mycelium	[48]
*A. niger*	Glucoamylase	Aeration and agitation intensities	Less compact surface structure of the pellets	[49]
*A. niger*	Fructofuranosidase	Osmolality	Dimensionless morphology	[50]
*Trichoderma reesei*	Cellulase	Agitation	Pellet	[51]
*A. niger*	Fructofuranosidase	Titanate microparticles	Loose inner pellets	[36]
*A. niger*	β-Fructofuranosidase	Addition of talc micro particles	Freely dispersed mycelium	[52]
*Mortierella isabellina*	Lipid	Additions of different concentrations of magnesium silicate microparticles	Free dispersed mycelia	[53]
*R. oryzae*	Fumaric acid	Soybean meal hydrolysate as the nitrogen source	Uniformly dispersed mycelial clumps	[54]
*A. sojae*	β-Mannanase	Addition of talcum and aluminum oxide	Pellet/mycelium mixture	[55]
*A. niger*	Enniatin B	Addition of talcum microparticles	Diameter 50–150 μm	[23]
*Mucor rouxii*	Chitosan	Replacing supplementary nutrients with fungal extract	Purely filamentous	[56]
*A. terreus*	Lovastatin	Addition of magnesium silicate	Small dense pellets	[57]
*A. niger*	Lipase	N:C ratio and FeCl_3_	Dispersed fungal morphology	[58]

**Table 2:** Overview of development of metabolic engineering morphology of filamentous fungi.

Strains	Products	Genotype	Morphology	Reference
*A. oryzae*	Amylase	Disrupt *chsB* and *csmA*	higher branch	[60]
*A. oryzae*	--	Overexpress *sclR*	extremely branched aerial hyphae	[63]
*Penicillium chrysogenum*	Penicillin	Class III chitin synthase gene silencing	shorter and more branched hyphae	[64]
*A. niger*	Protein	Lacks the gene related with the initiation of asexual sporulation	propagation through hyphae	[65]
*A. glaucus*	Aspergiolide A	Δ*AgkipA* mutant	pellets	[66]
*A. niger*	Citric acid	*chsC* gene silencing	pellets	[67]
*A. oryzae*	L-malate	Optimizing the expression of CDC14, CDC20 and CDC45.	pellets	[68]
*Aspergillus* species	--	Deficient in α-1,3-glucan and galactosaminogalactan	dispersed hyphae	[20]
